# Gluteal-fold flap repair of rectovaginal fistula caused by aluminum potassium sulfate hydrate-tannic acid injection for internal hemorrhoids: a case report

**DOI:** 10.1186/s40792-020-00925-7

**Published:** 2020-07-08

**Authors:** Masanori Yoshimitsu, Hiroyuki Egi, Shogo Nagamatsu, Manabu Shimomura, Keishi Hakoda, Masashi Miguchi, Toshihiko Kohashi, Masazumi Okajima, Hideki Ohdan

**Affiliations:** 1 Department of Surgery, Hiroshima City Hiroshima Citizens Hospital, 7-33 Motomachi Naka-Ku, Hiroshima, 730-8518 Japan; 2 Department of Gastroenterological and Transplant Surgery, Applied Life Sciences, Institute of Biomedical & Health Sciences, Hiroshima University, Hiroshima, Japan; 3 Department of Plastic Surgery, Hiroshima University, Hiroshima, Japan; 4 Department of Surgery, Hiroshima City Asa Citizens Hospital, Hiroshima, Japan; 5 Department of Surgery, Hiroshima Prefectural Hospital, Hiroshima, Japan

**Keywords:** ALTA, Internal hemorrhoids, RVF, Gluteal-fold flap

## Abstract

**Background:**

Rectovaginal fistula (RVF) following aluminum potassium sulfate hydrate-tannic acid (ALTA) injection therapy for hemorrhoids is a rare complication. We report the first case of RVF after ALTA injection therapy successfully treated by gluteal-fold flap.

**Case presentation:**

A 49-year-old female suffered from a fever and rectal ulcer after undergoing internal hemorrhoid treatment with a submucosal ALTA injection at a previous clinic. One week after ALTA therapy, she noted obvious passage of flatus and stool through the vagina, and was diagnosed with RVF by anoscope at another clinic. She was referred to our hospital 3 weeks after ALTA therapy. Sigmoid colostomy was performed for fecal diversion as a preliminary step for fistula repair. However, the fistula was scarred and the defect between the rectum and vagina did not improve at all. Ten months after ALTA therapy, we performed fistula repair by gluteal-fold flap. Seven months later, sigmoid-colostomy reversal was performed. The patient has experienced no RVF in the 3 years since sigmoid-colostomy reversal.

**Conclusions:**

The gluteal-fold flap strategy is a useful option for severe RVF management.

## Background

Rectovaginal fistulas (RVF) are abnormal communications between the rectum and vagina. RVF may be caused by obstetric trauma, inflammatory bowel disease, radiation proctitis, and rectal operations such as stapled anterior resections. In this case, RVF occurred following aluminum potassium sulfate hydrate and tannic acid (ALTA) therapy for internal hemorrhoids. ALTA (Zion, Mitsubishi Pharma Corp., Osaka, Japan) is a new sclerosant developed in Japan [1, 2]. ALTA therapy is effective for shrinking and hardening the hemorrhoids in order to eliminate prolapse and bleeding. Reported post-operative complications have included a slight fever, ischuria, anal pain, and rectal ulcer, most of which improved through conservative treatment [3–5]. However, in rare cases, RVF has been reported following ALTA therapy. Various surgical methods to repair RVF have been reported, but there are no clear guidelines regarding the management of these fistulas [6, 7]. This is the first reported case of successful use of a gluteal-fold flap to repair a severe RVF after ALTA therapy for hemorrhoids.

## Case presentation

**Fig. 1:**
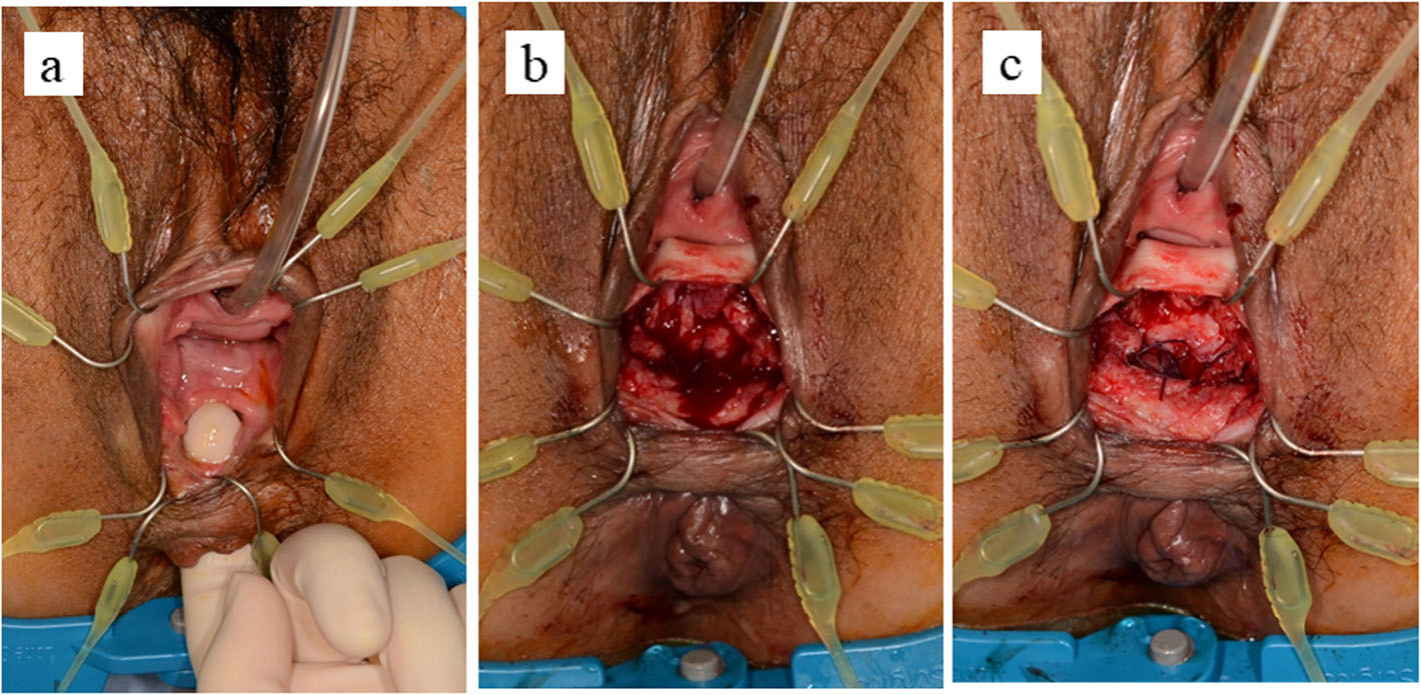
Intraoperative views of the rectovaginal fistula. (**a**) Rectovaginal fistula before repair; (**b**) after fistulectomy; (**c**) the rectal wall defect is closed and the vaginal defect is opened

A 49-year-old female suffered from a fever and rectal ulcer after undergoing internal hemorrhoid treatment with a submucosal injection of ALTA at a previous clinic. Detailed information concerning the ALTA therapy was not available. One week after ALTA therapy, she noted the obvious passage of flatus and stool through the vagina. She was diagnosed with RVF by anoscope in another clinic and referred to our hospital 3 weeks after ALTA therapy. A sigmoid colostomy was performed for fecal diversion as a preliminary step for fistula repair. The fistula did not improve at all (Fig. 1a). She had no symptoms such as pain and fever caused by severe damage of the surrounding tissue. However, she had been suffering from poor QOL as an ostomate. Ten months after ALTA therapy, we performed RVF repair by gluteal-fold flap. Under general anesthesia, the patient was placed in the lithotomy position. The 2 cm fistula at the vaginal entrance was easy to manipulate under direct vision. A fistulectomy was performed to dissect the fistula tract and the circumferential scar tissue (Fig. 1b). The rectal defect was closed primarily with interrupted absorbable sutures through the vaginal side. The vaginal wall was not sutured, and the space above the sutured rectal wall was opened (Fig. 1c).

**Fig. 2:**
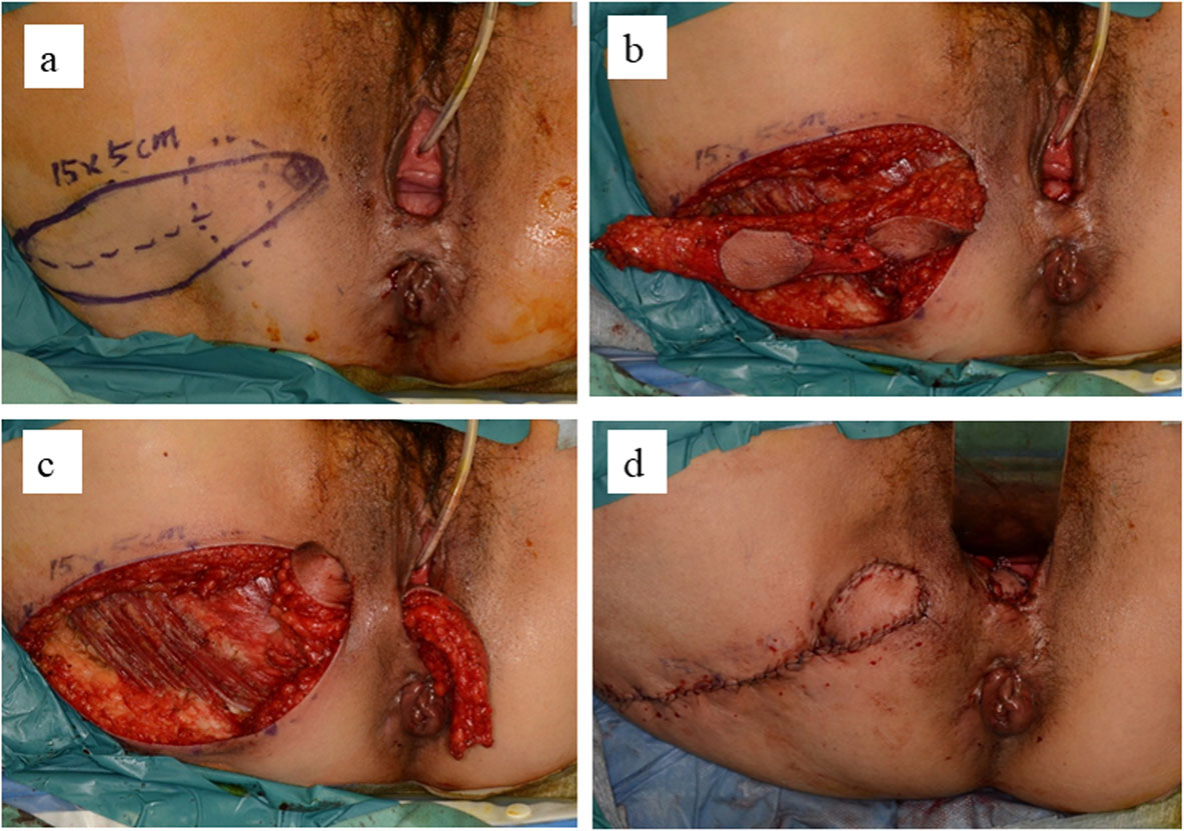
Intraoperative views of the gluteal fold flap. **a** Perforator vessels of the internal pudendal artery are identified with a Doppler probe and marked on the medial point of the right ischial tubercle. A 5 × 15 cm diamond-shaped flap of skin, subcutaneous fat, and superficial fascia is designed. **b** Perforators of the internal pudendal artery in the fatty tissue act as a pedicle and provide skin flap circulation. **c** The gluteal-fold flap is rotated counter-clockwise through a subcutaneous tunnel and placed in the space above the rectal wall. **d** The donor site is closed and the skin flap is sutured to the vaginal wall defect. The skin flap is observed in the vaginal vestibule

**Fig. 3:**
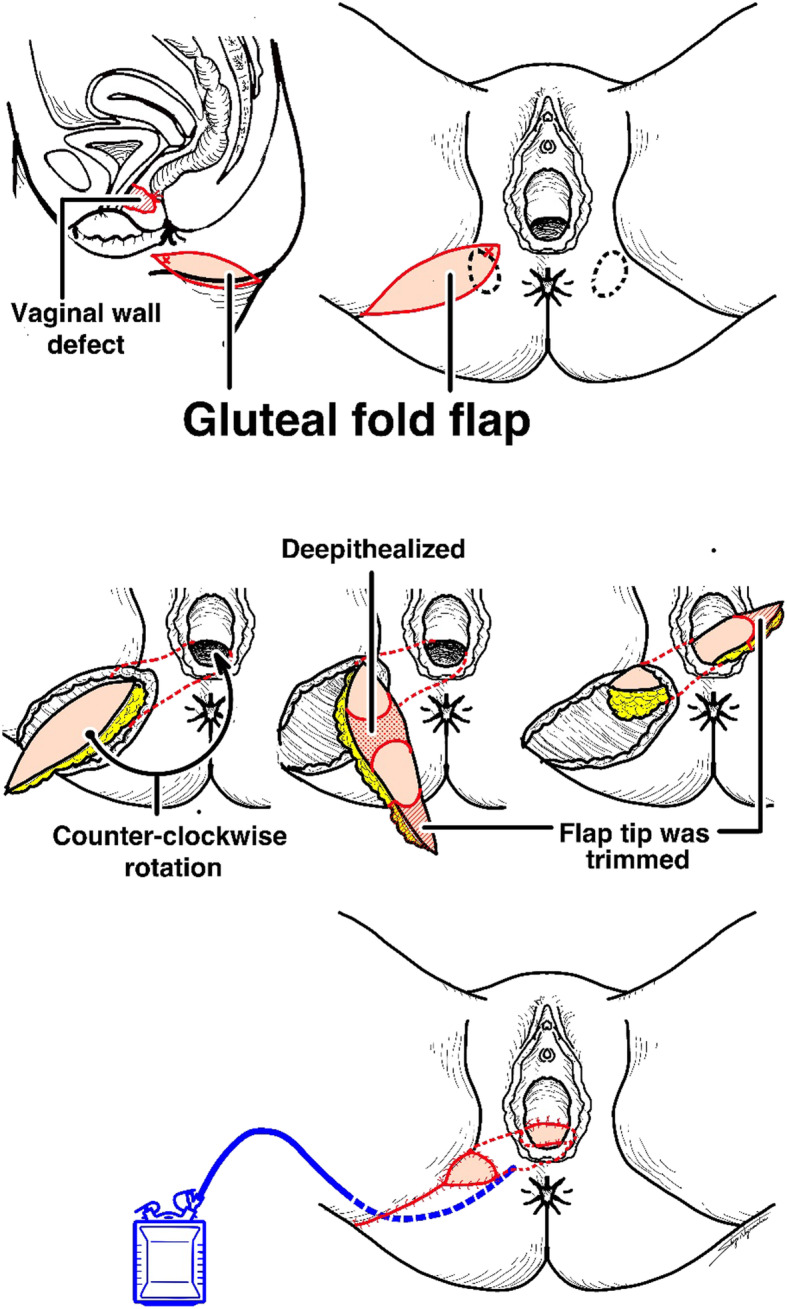
The illustrations of the operative procedure

**Fig. 4:**
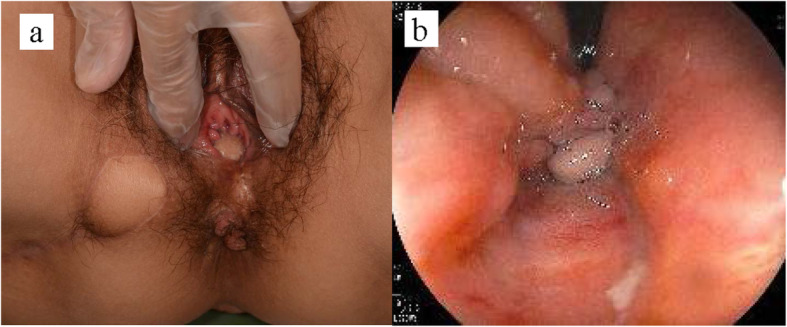
The healed site 6 months after the operation. **a** The gluteal-fold flap is seen in the vaginal vestibule with no fistula recurrence. **b** Colonoscopy shows no recurrence on the anorectal wall side

A Doppler probe was used to identify and mark the points of the perforator vessels of the internal pudendal artery on the medial point of the right ischial tubercle. A gluteal-fold flap was designed around the right gluteal fold including these marked points (Fig. 2a), the entire circumference of the skin was incised, and the distal side of the flap was elevated from the subcutaneous muscular layer. The proximal subcutaneous flap fat under the Doppler marking was left attached to the muscle to avoid injury to the pedicle vessel (Fig. 2b). After confirming the pedicle vessel of the flap, the flap was carefully elevated with blunt dissection. A subcutaneous tunnel to the vaginal introitus was prepared to minimize tension on the gluteal flap, and the flap was rotated 180 degrees and transferred through the tunnel to cover the posterior vaginal mucosal defect. The proximal skin portion of the flap was denuded and conformed to the shape of the posterior vaginal mucosal defect, and the subcutaneous fat tissue on the distal area of the flap was removed to thin the flap, while confirming bleeding from the flap margin (Fig. 2c). The flap was sutured to the surrounding vaginal wall with absorbable interrupted horizontal mattress sutures (Fig. 2d). Before donor site skin closure, a small suction drain was placed in the gracilis muscle harvested space. We show the illustrations of the operative procedure in Fig. 3. The patient had open drainage for subcutaneous abscess and was discharged on the 21st postoperative day. Seven months after the radical operation, we confirmed the fistula repair by pelvic examination of the vaginal wall and colonoscopic exam of the anorectal wall (Fig. 4). Sigmoid-colostomy reversal was performed. The patient has experienced no RVF and complete anal function without soiling and has not been trying to do sexual intercourse in the three years since the sigmoid-colostomy reversal.

## Discussion

This is the first reported case of successful gluteal-fold flap repair of severe RVF caused by ALTA therapy for the treatment of internal hemorrhoids. While iatrogenic RVF is known to be a rare, debilitating complication following the treatment of rectal cancer, there are no reports of refractory RVF following ALTA therapy in medical databases such as PubMed. These fistulas do not have the propensity to heal spontaneously and are challenging to repair. The fistula in this case was further complicated as it was fixed with scar tissue. Various surgical methods to repair these fistulas have been reported. However, RVF repairs sometimes break down when patients undergo multiple repairs [7, 8]. The gluteal-fold flap is one of the various muscle and skin flap repairs for the treatment of fistulas. A gluteal-fold flap is sufficiently wide and long to ensure adequate vascular supply and good mobility, providing a reliable surgical treatment for patients with severe and iatrogenic RVF [8–10].

ALTA, a new and effective sclerosant, was developed in Japan and is effective for shrinking and hardening hemorrhoids in order to eliminate prolapse and bleeding [1, 2]. ALTA therapy combines aluminum potassium sulfate and tannic acid; the former induces an inflammatory reaction, resulting in strong, local fibrosis, while the latter has a strong astringent effect on tissue, promoting protein coagulation and the contraction of blood vessels while reducing exudation into the tissue from the inflammatory reaction [1, 2]. ALTA sclerotherapy is popular as a comparatively simple, minimally invasive treatment for symptomatic internal hemorrhoids in outpatient clinics in Japan [3–5]. The symptoms of concern vanish practically on the first post-operative day. Patients usually experience little postoperative pain and no serious complications, and they report high satisfaction. ALTA therapy can be performed for patients suffering from Goligher’s grade II and III hemorrhoids in an outpatient setting [3].

While several reports have described post-operative complications including a slight fever, ischuria, anal pain, and rectal ulcer, most cases improved through conservative treatment, and no serious or life-threatening complications occurred [3–5]. However, ALTA damages tissue, and complications such as rectal ulcer and rectal stenosis have been reported due to the misplacement of injections [5]. In this case, detailed information from the previous clinic about the ALTA injection procedure could not be obtained. However, we guessed the RVF was caused by ALTA injection in vaginal side. The ALTA injection in front side requires close attention. Of note, this is the only local Japanese clinic to report a case of RVF associated with ALTA therapy for internal hemorrhoids. Conservative treatment, such as diverting ileostomy, has proven effective in treating RVF. However, in this case, the severe, scarred fistula could not heal spontaneously. Consequently, a challenging surgical repair was required.

## Conclusions

This is the first reported case of severe RVF caused by ALTA therapy for the treatment of internal hemorrhoids and successfully managed by gluteal-fold flap. RVF is a very rare and refractory complication of ALTA therapy that may be associated with misplaced injections. The gluteal-fold flap method used in the present case is a useful option for severe RVF management.

## Data Availability

The data presented in this article is not publicly available due to patient privacy concerns.

## Abbreviations

ALTA: Aluminum potassium sulfate hydrate-tannic acid
RVF: Rectovaginal fistula
